# Spatial and temporal proximity as factors in shape recognition

**DOI:** 10.1186/1744-9081-3-27

**Published:** 2007-06-05

**Authors:** Ernest Greene

**Affiliations:** 1 Laboratory for Neurometric Research, Department of Psychology, University of Southern California, Los Angeles, CA 90089-1061, USA; 2 Neuropsychology Foundation, Sun Valley, CA 91353, USA

## Abstract

Prior research from this laboratory examined minimal stimulus conditions that allow for recognition of objects. Using briefly flashed dots that marked the outer border of objects, it was found that timing differentials within and among successive dot pairs affected recognition, with significant declines being seen by the addition of temporal separations in the millisecond range. These experiments were done with dot pairs that had close spatial proximity, which leaves open the possibility that the effects could be attributed to strictly local neural encoding processes. The present research reports that spatial separation of pair members resulted in declines in recognition that were similar to those produced with close spacing of pair members. Both for close and separated dot pairs, recognition was best when they were displayed with near simultaneity, which likely generated synchronized spikes in the retina. These results provide cognitive evidence in support of proposals that synchronous neural activity is part of the image encoding process. The physiological literature is surveyed and discussed in an effort to delineate the issues, and a tentative model of retinal response to these stimulus conditions is offered.

## Background

"The conduction of one nerve tract is not insulated from that taking place in the remaining tracts. Hence there is a Gestalt relationship from retina on into the central fields." Kohler [1]

The amount of information that is provided by the image of an object is generally far more than is needed for recognition. One can eliminate many stimulus attributes, such as color and texture, and an observer likely will identify the object nonetheless. Further, a great many objects can be named when all of the internal features have been deleted, leaving only a silhouette boundary. It may come as a surprise, however, to learn that recognition is possible where very little information is provided with respect to the boundary, and with brief display of these minimal cues. Previous research from this laboratory has found that one can replace the outer boundary of an object with a spaced set of dots, each being shown successively for only a tenth of a millisecond, and subjects can still name the object at a level that is far above chance [2,3]. This is designated as the minimal transient discrete cue (MTDC) protocol.

Further, differentials in timing in the display of successive dot pairs greatly affects recognition. If the pairs are presented with fairly close spatial proximity of pair-members but with choice of location for successive pairs being random, the level of recognition is significantly affected by time differentials in the millisecond range. Providing as little as 2 ms of separation between successive pairs can produce a significant drop in recognition [2]. Further, providing as little as 0.5 ms of separation between the members of each pair will also result in a significant decline in recognition [2].

These results argue that the elements of a stimulus pattern, when shown very briefly, can access memory stores more effectively if they are displayed simultaneously or with timing differentials that are kept in the millisecond range. One possible explanation is that the brief stimulus pulses elicit synchronized firing that binds or otherwise distinguishes the shape cues that are needed to access memory [4-9].

One cannot assume that simultaneity of stimulus display will automatically generate synchronized neural activity. In the retina, where the neural encoding begins, the stimulus must traverse five major layers, requiring 15–35 ms in primates before it is converted into spikes that are sent up the optic nerve [10]. There are large differentials in ganglion cell response time that might be due to noise or unspecified internal dynamics. Further, even where synchronized firing has been documented (most of the work being in salamanders, rabbits and cats), most often it is identified on the basis of spike correlations in the non-stimulated retina, or in response to brightness transitions in a stimulus that extends across much or all of the retina, *i.e*., "full-field flicker." As reviewed more completely in the Discussion section, Meister and associates have argued that millisecond level synchrony in the firing of retinal ganglion cells is produced when there is simultaneous activation of overlapping receptive fields.

The Greene [2] study used successive pairs that had close spatial proximity of the pair members, the kind of stimulus that would likely stimulate overlapping receptive fields. It would be useful to know whether this proximity is essential. The present research used the MTDC protocol to both replicate the critical finding that very brief delays can produce major deficits in recognition, and to examine the importance of spatial proximity of stimulus elements. Briefly anticipating the results, the two experiments reported here also found that time-delays in the millisecond range among and within pair members impaired recognition of objects. Spatial separation of the pair members produced a modest penalty in recognition, but the decline in recognition as a function of temporal proximity was similar to that found for adjacent pairs. These results complement recent reports of synchrony in retinal ganglion cells that are well separated, and supports the conjecture that correlated neural activity in the retina is involved in encoding of shape contours.

## Methods

Twenty four subjects were tested, twelve for each of the two experiments using an MTDC protocol (as detailed below). Stimulus sets were the same for both experiments, as were task conditions except for timing differentials for display of successive dots.

Each shape was suggested by a set of dots that marked locations at the boundary of the object, which is described as display of a "shape pattern." The number of dots displayed for a given shape pattern was designed to provide minimal cues for recognition of the object, thus reducing recognition into a range below 80% so that the effect of timing differentials could be examined.

One hundred fifty shape patterns were displayed to each subject, this inventory being provided in Table 1 of Greene [2]. A given shape was shown to the subject only once. Each shape pattern had been sized and discretized so that the horizontal or vertical dimension of each shape fit to the edges of a 64 × 64 grid. The locations at which the outer boundary of a given shape crossed the cells of the grid provided an address table of boundary locations, and this table was subsequently sampled to specify the positions on a 64 × 64 LED display board that were activated to display each shape pattern.

The shape patterns were shown on the display board under the control of a G-4 Mac computer and microprocessor slave. The LEDs of the display board emitted at 660 nm, with rise and fall times of less than 100 ns and with a luminance of 10 Cd/m^2^. Room illumination was from standard ceiling-mounted fluorescent fixtures that were fitted with opaque panels to block most of the light. This provided ambient illumination of 13.3 lux, which yielded luminance from the wall against which the display board was mounted of 1 Cd/m^2^.

Subjects sat across the room from the display board, being allowed free binocular viewing at a distance of 3.5 m. At this distance the full spans of the LED array were 7.7 by 7.7 arc°, center-to-center spacing among LEDs was 7.4 arc', and the diameter of each LED was 4.9 arc'.

**Figure 1:**
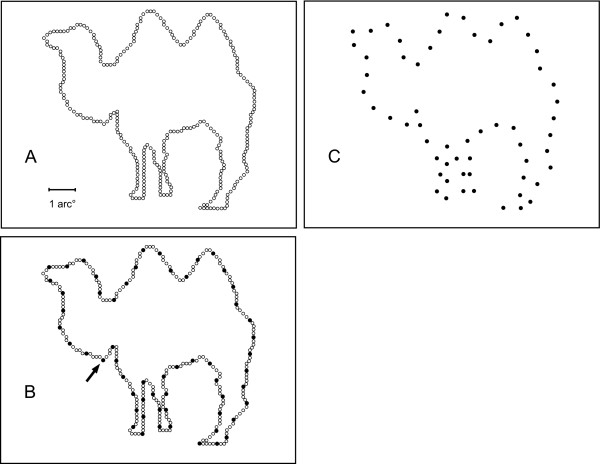
A. Positions of the boundary markers for a typical shape are illustrated. A size bar representing 1 degree of visual angle is provided in the lower left corner of this panel. B. A sample from the full inventory was selected for display. For a given subject a random starting point was chosen, designated here by the arrow. Then proceeding clockwise, every Nth dot was selected to be in the display set. The value of N varied across shapes to adjust for difficulty. C. The dots of the display set are shown. Dot size has been enlarged for purposes of illustration.

For a given subject, a sample was chosen from the address table of each shape to be the display set for that shape. Figure 1 illustrates the method for choosing the display set using the camel shape, for which the number of dots in the set matched the average across all shapes in the inventory. Beginning at a randomly chosen location within the address table, every Nth address (dot) was included in the sample, with the value of N ranging from 3 to 10. The value of N for a given shape, designated as the skip factor, was based on prior testing that established what spacing would provide equally recognizable shape patterns. The spacing was expected to produce approximately 75% success in recognition if all dots in the display set were shown successively with a duration of 0.1 ms, and with offset of one dot being followed immediately by onset of the next.

A major experimental question was whether the spatial proximity of successive dots influenced the recognition potential of the shape patterns. This was examined by selecting pairs of dots from the display set, requiring either that the members of the pair be adjacent in the sequence of display set addresses, or as far apart as possible in this sequence. These two levels of spatial proximity were designated as "adjacent" and "opposite" pairing of the dots, respectively.

**Figure 2:**
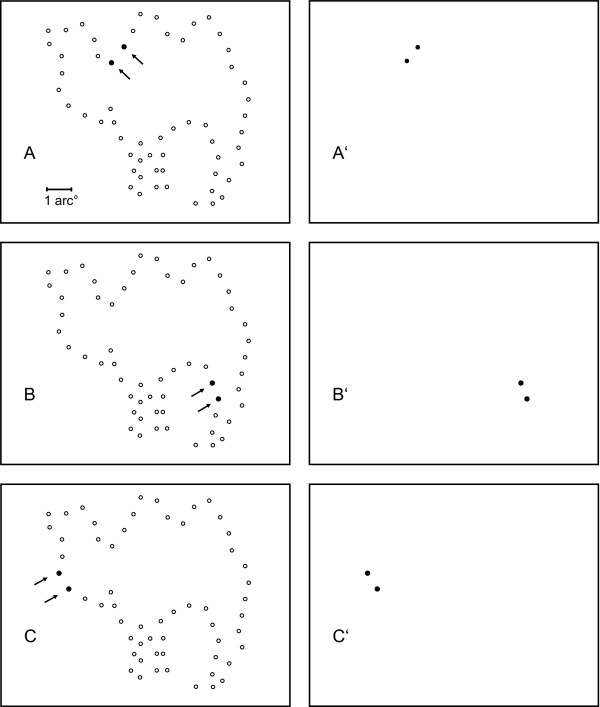
The left panels illustrate selection of dot pairs for the adjacent condition. Spatially contiguous pairs were selected from random locations to be displayed successively. The right panels show each pair being flashed, with successive pairs being shown at A', B' and C'.

**Figure 3:**
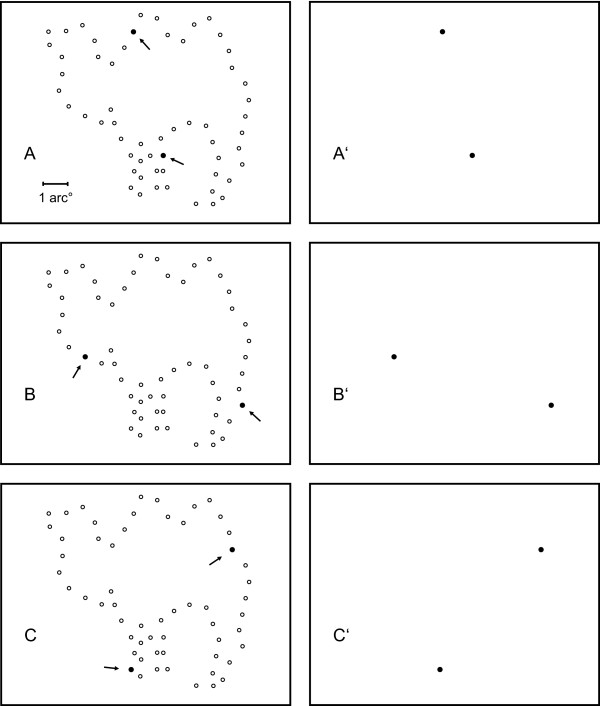
The panels of Figure 3 show selection and display of pairs having greater spatial separation. This was designed as the "opposite" condition, meaning that the pairs were selected from the full inventory of addresses, and requiring that they be as far apart as possible in the address list.

For a given shape, the order in which pairs were displayed was chosen at random. Figures 2 and 3 illustrate the protocol. The left panels of Figure 2 show the choice of four successive pairs of "adjacent" dots (filled circles) in relation to the full inventory of addresses that were not sampled (open circles). Note that the skip factor has been applied, so the term "adjacent" relates to the successive positions in the display set, not the full inventory of addresses. The right panels of Figure 2 illustrate display of these dots on the otherwise blank, non-emitting, array of the LED board. Adjacent pairs from the display set were selected at random until all pairs in that set had been shown, after which any remaining unpaired dot was displayed. Figure 3 provides the same illustration with respect to pairs from the "opposite" treatment condition.

**Figure 4:**
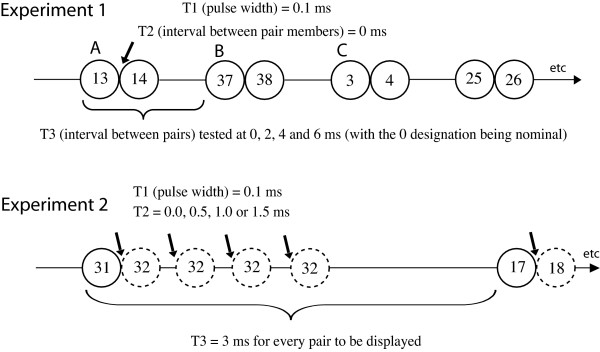
Timing conditions for the two experiments are illustrated. The first three dot pairs in the upper sequence correspond to the samples that were illustrated in Figure 2, panels A, B and C, respectively. Each dot in the display set was flashed only once for 0.1 ms, this being T1. T2 was the interval from offset of one member of the pair till onset of the other member. For Exp. 1 this was 0 ms, and for Exp. 2 the value varied between 0 and 1.5 ms. T3 was the interval between successive pairs. In Exp. 1 it varied between 0 and 6 ms, and in Exp 2 it was kept constant at 3 ms.

Timing conditions for display of the dot patterns in the two experiments are illustrated in Figure 4, and here it will be convenient to describe the flash from a given LED as a pulse. Pulse duration was 0.1 ms for each dot that was displayed, this being designated as T1. Temporal separation of dot pairs, designated as T2, was measured from offset of the first member of the pair till onset of the second member of the pair. Separation of one pair from the next was measured from onset of one pair till onset of the next, this being designated as T3.

For Experiment 1, all shapes were displayed with a T2 = 0, and four levels of T3 were tested, these being 0, 2, 4 and 6 ms (with 0 being nominal, the actual value being 0.1 ms). Experiment 2 tested with four levels of T2, these being 0.0, 0.5, 1.0 and 1.5 ms, with T3 being held constant at 3 ms.

There were eight treatment combinations in each of the two experiments. Specifically, in Experiment 1 the two levels of dot proximity (adjacent vs. opposite) were combined across four levels of T3 interval (0, 2, 4 and 6 ms). For Experiment 2 there were the two levels of dot proximity combined across four levels of T2 (0.0, 0.5, 1.0 and 1.5 ms). A given subject saw each of the 150 shapes only once, each shape having been randomly assigned to one of the treatment combinations, and being shown in random order. This was not a speeded task, but subjects generally gave a response within a second or two following display of the shape pattern. The experimenter recorded whether or not the answers were satisfactory without knowing what treatment combination had been used for display of the shape pattern(s).

## Results

The subject either correctly identified a given shape, or did not, these being binary alternatives. The proper model for such data is a generalized linear mixed model that treats the binomial errors using a logit link function [11]. This is used because with only two possible values for each judgment, *i.e*., 0 or 1, the residuals in a statistical analysis will not be normally distributed. However, log transform of the proportion ratios, *i.e*., (log_e _(proportion/1 – proportion) – known as logit values – provides corrected data that are normally distributed. For this analysis, logit values were derived for each treatment combination, and these were compared using the standard error of the difference for these values. Shape and subject variables were treated as random effects. The number of dots displayed for a given shape was included as a variate for each analysis.

For Experiment 1, proximity of pair members, *i.e*., adjacent vs. opposite, and the four levels of T3 (0, 2, 4 and 6 ms) were treated as fixed effects. Mean logit scores for the adjacent and opposite treatment conditions were 0.587 and 0.253 respectively, with the standard error of the difference being 0.106. The treatments were significantly different at p < .01. These logit means correspond to back-transformed predictions of 64% successful recognition for the adjacent-paired condition and a 56% level of recognition for the opposite-paired condition.

**Figure 5:**
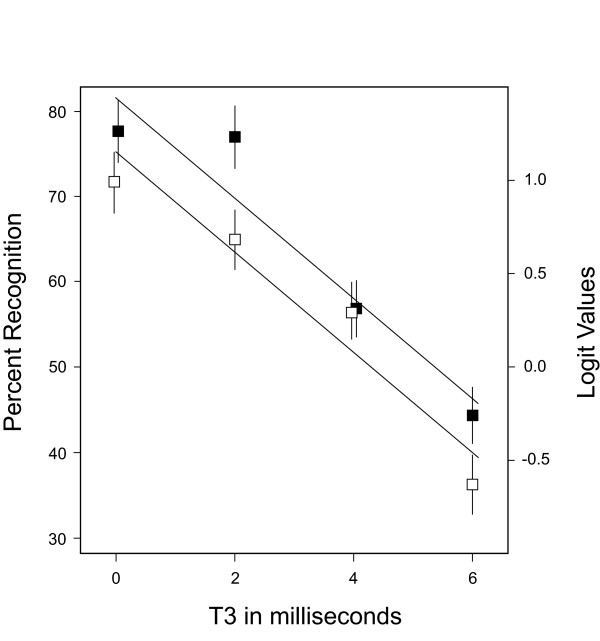
In Exp. 1, recognition was in the 70% range at T3 = 0 ms, and the hit rate then declined as a function of the T3 interval. Adjacent means are plotted using filled squares, and opposite means are plotted using open squares. Although there was a significant differential for the spatial proximity conditions, with opposite pairs consistently scoring below adjacent pairs, the pattern of decline was the same for both treatments. Linear regression lines have been plotted, based partly on prior research that showed the decline to be linear with task and stimulus conditions that were identical to the adjacent condition of the present study.

There was a significant (p < .001) decline in recognition as a function of the T3 interval that dropped from the 70% range into the 40% range for both adjacent and opposite pairing conditions. There was no indication of an interaction with respect to T3. The predicted means from the generalized mixed model are plotted in Figure 5, along with the plus and minus error bars indicating the size of the standard error for each mean. These error bars should be interpreted against the logit scale that is on the right. A unit increase in T3 corresponded to the odds of recognition being multiplied by a factor of 0.80 (95% CL = 0.76, 0.83).

A prior report [2] used an adjacent pairing protocol that was essentially the same as for the present research, and found clear evidence that recognition dropped as a linear function of T3 interval. In the present experiment the hit rate for the adjacent condition is well above the linear regression line at 2 ms, and several of the opposite pairing means do not fit the linear regression line very well. Indeed, for both proximity conditions a regression model that included a quadratic component would provide a better fit to the data.

For Experiment 2, the two proximity conditions (adjacent/opposite) and the four levels of T2 (0.0, 0.5, 1.0 and 1.5 ms) were treated as fixed effects. Mean logit values for the adjacent and opposite treatment conditions were -0.108 and -0.373, respectively, with a standard error of the difference being 0.092. This difference was significant at p < .001. The mean logit values correspond to predicted levels of percent recognition of 47% and 41% for the adjacent and opposite treatment conditions, respectively.

**Figure 6:**
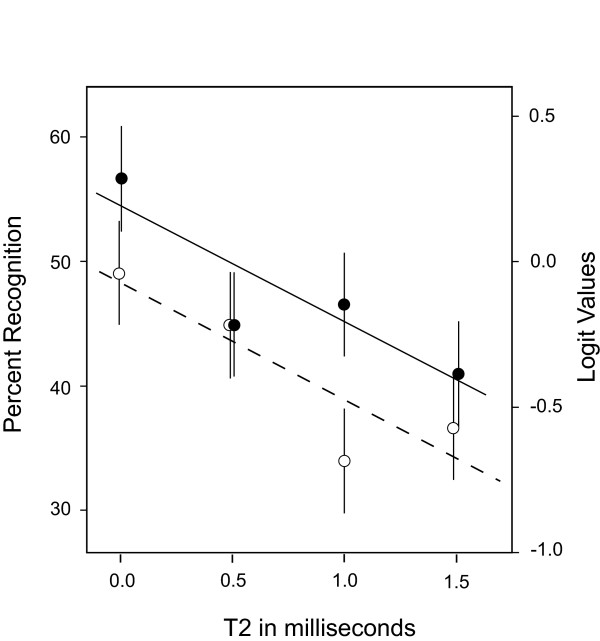
For Exp. 2, adjacent means are plotted as filled circles, and opposite means are plotted as open circles. Here also there was consistently lower recognition for the opposite condition, but recognition declined as a linear function of T2 interval for both, and there was no evidence of a differential effect, *i.e*., interaction, as T2 was varied.

Predicted means from the generalized mixed model are plotted in Figure 6, along with the plus and minus standard error bars and regression lines. There was a significant decline in recognition as a function of the T2 interval (p < .001), with no indication of a nonlinear component or interaction as a function of pair-proximity. A unit increase in T2 corresponded to the odds of recognition being multiplied by a factor of 0.43 (95% CL = 0.25, 0.76). As was found in earlier work [2], it is especially notable that the declline observed at T2 = 0.5 ms differed significantly from the recognition levels at T2 = 0. This again affirms that the boundary dots are more effective as shape cues if they are yoked by simultaneity in the submillisecond range.

From the data plots in Figures 5 and 6, it might appear that T3 had a stronger influence on recognition than did T2. This is not the case if one evaluates the strength of effect on the basis of how each millisecond of temporal separation affected recognition. Measures taken from the regression line are adequate for purposes of discussion, with T3 showing a 6% drop in recognition level for each millisecond of temporal separation, and with the T2 drop being 10% per millisecond. Although the difference in effect size is not as large as was found previously [2], it does affirm the strong impairment that is produced by very brief delays in the stimulus sequence.

As outlined in Methods, the spacing of dots (the skip factor) was chosen to provide for approximately equal probability of recognition if all the dots were shown as rapidly as possible, i.e., T3 = 0. Providing a number of levels of T3 introduced the possibility of a differential potential for recognition as a function of the number of dots being displayed, therefore Dot# was included as a variate in the analysis.

**Figure 7:**
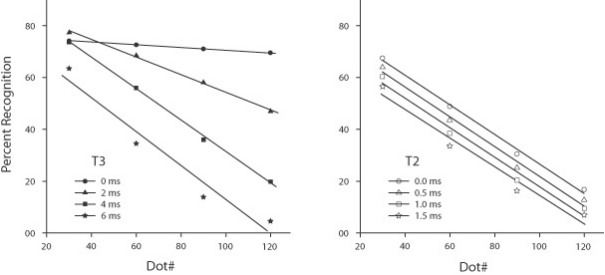
Recognition was a joint function of the number of dots in the display sets (Dot#) and T3 intervals. For Experiment 1 (left panel) the plot is fairly level where T3 = 0 ms, but the slope drops and grows progressively steeper as the T3 interval increases. The plots for Experiment 2 (right panel) show declines as a function of Dot# where T3 = 3 ms, with the T2 interval providing equivalent, and thus parallel, contributions to recognition across the full range of Dot#.

For Experiment 1, Dot# also produced a significant decline in recognition (p < .001), and there was a significant interaction between T3 and Dot# (p < .001). These effects are plotted in the left panel of Figure 7. For this figure, the logit predictions for Dot# = 30, 60, 90 and 120 have been backtransformed into probability scores, and a regression line has been fitted to these values. The plot for T3 = 0 ms is relatively flat across the full range of Dot#, which affirms the criterion by which the skip factor was chosen. A slope of the regression line drops and becomes progressively steeper as T3 is increased to 2 and then 4 ms, but the slopes for 4 and 6 ms are approximately the same. This suggests that the source of interaction between T3 and Dot# has reached its limit of contribution between 2 and 4 ms, as further evaluated below.

The analysis of Experiment 2 also examined how Dot# affected recognition where T2 was varied, with T3 being 3 ms for each shape that was displayed. The plots in the right panel of Figure 7 show that Dot# produced a significant decline in recognition (p < .001), this being attributable to the 3 ms separation between successive pairs. However, there was no indication of an interaction of T2 with Dot# (p = .32). The near parallel relationship among the regression lines affirms that the T2 interval contributes equally to recognition irrespective of the size of the display set.

Separation of dot pairs produced a lower per millisecond decline in recognition than did separation of pair members. A differential in the slope might be produced by a single mechanism with a temporal decay curve that yielded strong linkage of dots at very short intervals and weak linkage at much longer intervals. However, the parallel structure of the T2 effect, shown in the right panel of Figure 7, suggests that T2 effects do not differ as a function of the number of dots. This argues that the T2 and T3 intervals affect two separate encoding and/or recognition processes.

It is possible that T2 levels of simultaneity provide for the interaction that can be seen in the left panel of Figure 7, which ostensibly plots T3 effects. Here we evaluated T2 from 0 to 1.5 ms, but the system that is sensitive to brief time differentials could well be registering simultaneity over intervals of several milliseconds. If so, then the T3 interval of 2 ms would enhance recognition in the same manner as small differentials in the T2 interval. The parallel regression lines for T3 equal to 4 and 6 ms suggests that this requirement for simultaneity, what might be called the T2 domain, does not extent to these longer intervals.

One might wonder whether the T3 effect for pair separations beyond 4 ms should be attributed to the total time required to show the display set rather than the time differentials among successive pairs, *per se*. Perhaps there is a temporal integration window that combines all dots that have been displayed within a specified interval, and level of recognition depends on whether the T3 interval is short enough to get all (or most) of the dots into the window. This is the major thrust of the iconic memory/visible persistence literature, which has been treated in greater depth elsewhere [3]. Here it may be adequate to note that the results plotted in Figure 7 are not consistent with a fixed integration window concept. For a given interval that might mediate integration, one would expect recognition to be relatively flat for the Dot# that could be delivered during that interval, and to decline only when the interval was exceeded. One can see that for each level of T3 – 2, 4 and 6 ms in the left panel, and 3 ms in the right panel – there is a smooth decline across the full range of Dot#.

However, the width of the integration window could be related to the number of dots needed for recognition. The size of the display was adjusted for familiarity and complexity of the stimuli. Although this adjustment rendered the shapes approximately equal in their potential for recognition when the dots were shown quickly, i.e., T3 = 0, the memory register itself might be sensitive to total time of display. The integration window might be shorter or longer, depending on the intricacy and/or familiarity of the shape.

## Discussion

This research confirms the previous finding [2] that effective integration of briefly displayed border dots depends on the degree to which the dots are simultaneously presented. The studies indicate that millisecond and even submillisecond simultaneity in the display of border dots affects whether the dots will provide effective cues for recognition of shape patterns. The present results also show that the requirement for simultaneity is not limited to dots having close spatial proximity. These findings call for temporal precision in the encoding process, most likely generating synchronized spikes in retinal ganglion cells.

As indicated at the outset, one cannot assume that simultaneity of stimulation will be translated into synchronous firing of the ganglion cells that provide the optic nerve signal. Maunsell *et al*. [10] examined the latency of responses in lateral geniculate nucleus of Macaque, and found substantial variability in response latency to the onset of a high luminance spot. Maunsell & Gibson [12] report the shortest range of latencies for signals arriving at primary visual cortex in Macaque, these being from 15 to 55 ms. Primate data from several other laboratories show much higher variability, as summarized by Nowak & Bullier [13].

On the other hand, using full-field flicker, Uzzell & Chichilnisky [14] reported precision in the 1 ms range for primate ganglion cells [see also [15-18] for work in other species]. It is not clear whether the reports of high temporal precision with full-field flicker provide a solid base for inferences about linkage of discrete, spatially separated stimuli. Although cross-correlation of many ganglion cells with full-field flicker show a peak with 0 ms (nominal) synchrony, the half-width of the correlation is commonly in the range of 20 ms [19]. Further, most of the spikes are not time-locked to stimulus onsets [19], and a good portion of the synchronous activity that is produced may be due to simultaneous activation of overlapping receptive fields [20,21].

For the adjacent condition of the present experiments, the spatial proximity of pair members could have been close enough to activate overlapping receptive fields. Every fourth dot was chosen for display from the full inventory of addresses for the average shape. Horizontal and vertical separation of LEDs within the array was 7.4 arc'. Thus, if one ignores the differential from diagonal spans, the average separation of dot pairs was in the range of half a degree of visual angle. Most pairs would be placed away from the center of fixation by two degrees or more, and at this eccentricity one cannot rule out overlap of the receptive fields being stimulated by the pair. Given these factors, for the adjacent condition we cannot dismiss the possibility that synchronous ganglion cell firing was produced directly by joint activation of common, *i.e*., overlapping, receptive fields.

However, the present finding that simultaneity is important for widely spaced dot pairs suggests that synchrony of firing is possible without overlap of the receptive fields. Further, a number of studies have provided evidence for 0–1 ms synchronization of activity in retinal ganglion cells having large spatial separation, and some of it relates specifically to the encoding of contours. High frequency oscillatory responses in the retina can be triggered by a contour, even for receptive fields that are separated by up to 20 arc° [22,23]. These high-frequency oscillations could serve to control the precision of synchronous firing [24].

In support of that possibility, Amthor *et al*. [25] found synchronized firing in directionally sensitive retinal ganglion cells of rabbits when they were stimulated with a moving edge. These investigators did not find synchrony with a number of other stimulus conditions that produced high firing rates, including random full-field flicker. Correlations from small moving spots were relatively weak. The correlated activity could be elicited in cells with non-overlapping receptive fields, and between cells that coded for different directions of motion. For pairs of cells that had orthogonal direction preferences, the most effective stimulus was one that moved along an axis that was intermediate between the two preferences.

Further, Chatterjee *et al*. [26] used multielectrode arrays and found that several different classes of ganglion cells provided synchronized firing in response to moving bars and edges, and the correlated activity was observed with receptive field separations of up to 960 micrometers (7.4 arc°). Full-field flicker did not produce millisecond time-scale correlation within or across cell classes.

While the studies showing synchronized responding of ganglion cells [22-26] are encouraging, it should be remembered that their results have been produced using spatial extended and moving stimuli. Further, the reports of temporal precision are based on correlations across long intervals. Finding high temporal precision under these conditions does not provide direct evidence that similar close timing would be provided by dots that were flashed for 0.1 ms. On the other hand, it does not seem unreasonable to assume that neural systems that can generate spikes in response to submillisecond rise-time of electrical stimulation could produce synchronized firing in response to briefly flashed dots.

Highly synchronized responding to spatially separated stimulation might be coordinated by wide-field amacrine cells that can link ganglion cells by means of gap junctions [27-32]. Many amacrine cells extend across the retina for several millimeters [30,33,34], which in central vision would provide linkage cross the full span of the display field. Electrical coupling through gap junctions can provide temporal resolution that is shorter than what is provided by chemical transmission. Spread of neurobiotin indicates that parasol ganglion cells of macaque receive gap-junctions from such wide-field amacrine cells [35-37].

A number of investigators have suggested that synchronized activity of retinal ganglion cells serves to encode contours, and may be especially valuable for registering the boundaries of moving objects [22,25,26]. One might elaborate by suggesting that the evolution of shape perception began in primitive oceans, with movement of silhouettes providing the salient cues for deciding whether to feed or flee. Survival could depend on evolving special circuits to register the coordinated movement of these silhouette boundaries, which then extended to coincident stimulation by still boundaries as the creature swam. It would also need to track or detect a moving object that was passing behind seaweed or other sources of visual obstruction. Here it might have only a brief glimpse of spatially disconnected points in the boundary of the object, with coincidence – what Wertheimer [38] called "common fate" – providing the only evidence that these stimulus events were generated by a unified source. Recognition of objects using a minimal number of transient and spatially separated discrete cues could determine its ability to survive and reproduce.

## Conclusion

Whether or not synchronous activity provides a general principle for encoding shape contours, or has a more limited role, it is clear that objects can be recognized even with a minimal display of boundary markers, and with each of these being shown for a brief moment. Close temporal proximity of successive shape cues is needed for effective encoding of these cues. Spatial separation of pair members impairs recognition, but within the distances tested here, the same basic process appears to bind pairs that have been synchronously displayed irrespective of spatial proximity. With intervals of 4 ms and above, the decline in recognition may be due to the total time it takes to display all the dots. While the present results do not support the idea of an integration window of fixed duration, it is possible that the aggregate time required for recognition varies as a function of the number of dots needed for recognition.

## Abbreviations

arc° : degrees of visual angle

arc' : minutes of visual angle

Cd/m^2 : ^candela per meter squared

GaAlAs : gallium, aluminum and arsenic

LED : light emitting diode

Log_e _: natural log

lux : lumen per meter squared

m : meters

ms : milliseconds

MTDC : minimal transient discrete cue

N : number used to specify how many dots from address list will be displayed

nm : nanometers

ns : nanoseconds

p : probability

T1 : pulse width

T2 : temporal separation between members of subset pairs

T3 : temporal separation between subset pairs

## Competing interest statement

The author declares that he has no competing interests.
